# Identification of Seniors at Risk: transcultural adaptation for Brazilian Portuguese

**DOI:** 10.31744/einstein_journal/2022AO5705

**Published:** 2022-03-04

**Authors:** Thabata Cruz de Barros, Henrique Salmazo da Silva, Beatriz Aparecida Ozello Gutierrez

**Affiliations:** 1 Escola de Artes, Ciências e Humanidades Universidade de São Paulo São Paulo SP Brazil Escola de Artes, Ciências e Humanidades, Universidade de São Paulo, São Paulo, SP, Brazil.; 2 Universidade Católica de Brasília Brasília DF Brazil Universidade Católica de Brasília, Brasília, DF, Brazil.

**Keywords:** Aged, Integrality in health, Hospitalization, Risk management, Continuity of patient care

## Abstract

**Objective:**

To perform cross-cultural adaptation of the Identification of Seniors at Risk tool.

**Methods:**

This methodological study was based on the guidelines process proposed by Beaton, attending the stages of translation, back-translation, judgment by judges and content validation of the Identification of Seniors at Risk tool. The goal of this tool is to allow the identification of the elderly at risk for hospitalization, composed of six dichotomous questions (yes or no) related to functional decline, comorbidities, previous hospitalization (last 6 months), visual impairment, significant changes in memory and polypharmacy. Two bilingual translators and 16 health professionals with hospital and academic performance in the fields of geriatrics and gerontology participated in the study.

**Results:**

Differences were observed between the initial translations and the final version. Changes were made to questions 1, 3, 4 and 6. In the last question, an item was modified to meet the Brazilian polypharmacy criterion. After the cross-cultural adaptation, the tool showed 100% agreement between the judges.

**Conclusion:**

Brazilian Identification of Seniors at Risk has indexes of verbal comprehension and high content validity.

## INTRODUCTION

The demands associated with the population aging process have generated new challenges to social and health care. Among these challenges, there are the increase of elderly with chronic diseases, dependency and frailty, and decrease of physiological reserves.^(1)^

In the published literature, frailty is associated with lower functional performance, falls, hospitalization, and deaths.^(1)^To reverse this process, specific care planning, health care qualification, and the use of appropriate tools to detect adverse outcomes in the elderly population accessing the different health services are essential. This is particularly true since the elderly use of hospital services are more frequent than general population.^(2)^

In this approach, the adequate prediction of risks associated with the worsening of the elderly people’s health status may help to structure an effective, resolutive and optimized care. This would allow the appropriate allocate resources, avoidance of ineffective interventions, and reduction of recurrent hospitalizations.^(3)^

In the context of long-term care, hospitalization implies longer treatment, slower and more complex recovery, decreased functional capacity, and changes in the perception of quality of life. In this sense, to identify the factors that influence the occurrence of hospitalizations is essential among the elderly population, so that preventive actions and health promotion can be developed.^(2)^

Previous findings with sensitive and validated instruments have observed that the following constitute risk factors related to hospitalizations, *i.e*., aged over 75 years, male gender, no caregiver, self-perception of health as poor, report the presence of cardiovascular disease, presence of *diabetes mellitus*, and hospitalization within the last 12 months, as well the attendance to more than six medical visits within the last 12 months.^(4-7)^

Among the tools used in the published international literature, the Identification of Seniors at Risk (ISAR) is highlighted. The ISAR was developed in Canada by researchers at St. Mary’s Research Center and McGill University. It is a screening tool that can quickly identify seniors who are in need of a more complete or comprehensive assessment.^(8)^

The ISAR has six questions that can be answered by the patient themselves, by family members, or by professionals from different specialties. These six questions refer to the need reported by the patient for Activities of Daily Living before the time of hospitalization. If he/she will need help after hospitalization, problems related to visual acuity, perceived memory problems, number of hospitalizations in the last semester, and total number of medications per day. These questions have dichotomous answers (yes or no), and a score is assigned to each answer. A score above two represents that the elderly person in question is at risk, and, in such a situation, some referral suggestions are oriented by the authors. Among these suggestions there are the notification to family members, to the responsible physician for the patient, as well as the performance of an evaluation of the elderly patient by specialized professionals. In the original study the ISAR tool showed a sensitivity of 74% and specificity of 54% for scores higher than two points. In addition to these data, there is evidence of moderate predictive validity and a significant correlation for recurrent emergency department visits, hospitalizations, decreased functional capacity, and mortality.^(8)^

In 2015, a systematic review was published on the use of the ISAR conducted from 10 studies from January 1999 to December 2014, which found that the predictive validity of the ISAR was controversial. These studies were in English language. The total sample size was 8,680 patients, with a cutoff score of at least two. The ISAR was shown to have low validity related to patient return to the emergency room and hospital readmission.^(9)^ The predictive validity of the ISAR related to mortality was also found to be rated as poor to reasonable.^(9)^ In the end, it was concluded that the ISAR is limited in its predictive ability at cutoff point ≥2 points to detect elderly patients at risk of adverse health outcomes after emergency room care. For this reason, guidelines recommend that further studies should be conducted to evaluate the predictive ability of the ISAR.^(9)^

In 2016, a systematic review with meta-analysis, analyzing 32 articles that applied the ISAR in the emergency room, detected that the ISAR has moderate predictive accuracy as a screening tool in the emergency room, with consistently high estimates of pooled sensitivity across all outcomes and time points. It may be useful in clinical decision making in determining which older adults can be safely discharged from the emergency room.^(10)^

In terms of the need to identify the elderly at risk in the context of Brazilian emergency services, we identify that, in general, it is a challenging to have a tool that can collaborate in this work process, particularly, aiming to prevent adverse outcomes in elderly patients, and allow their selection for geriatric interventions and based on local organizational and economic resources.^(11-16)^

## OBJECTIVE

To perform cross-cultural adaptation of the Identification of Seniors at Risk tool.

## METHODS

This was a methodological study for the cross-cultural adaptation of the ISAR tool aiming at its use in elderly patients seen in emergency rooms. The investigation was conducted from April to June 2016 at the *Hospital das Clínicas da Universidade de* São Paulo (USP) in São Paulo (SP), Brazil.

The ISAR enables the identification of the elderly at risk based on closed answers (yes or no) to six questions related to functional decline, comorbidities, previous hospitalization (last 6 months), visual *deficit* that cannot be corrected with the use of glasses and corrective lenses, significant memory changes, and polypharmacy. It is noteworthy that this tool, developed to be used in emergency services, can be responded by elderly patients, caregiver or trained professional.^(10)^

Cross-cultural adaptation is an important process to ensure equivalence between the original language and the translation into the target language. Furthermore, this process enables the validation of the instrument in relation to its measurement standards, which may have different connotations in different cultures.^(17-19)^This aspect should also be respected for phenomena related to the health-disease process, since the local culture influences its perception and expression.^(18)^For this reason, the cross-cultural adaptation recommends the fulfillment of two stages, which are the translation and cultural adjustment of the instrument and its content validation.

In this context, this study used the method proposed by Beaton et al.,^(16)^that guides the process of cross-cultural adaptation based on five stages: translation of the instrument, synthesis, back translation, evaluation by a group of judges and pre-test.

The cross-cultural adaptation process of the ISAR for use in elderly patients admitted to the emergency department consisted initially of translations of the original ISAR into Brazilian Portuguese. Thus, in step 1, two translations (T1 and T2) of the ISAR were performed, as recommended by the guideline chosen for this research.^(17)^

For stage 1, two independent translators were invited, the first translator being a health professional with a nursing degree (responsible for version T1), and the second translator was a journalist (responsible for version T2). Both translators were fluent in English, and only the first translator (health professional) was informed about the objectives of this research.

From the two translations (T1 and T2), step 2 was developed, a synthesis version, resulting in translation 3 (T3).

Step 3, the back translation of T3, was carried out by two independent translators who had the same mother tongue of the original tool and a command of Portuguese, resulting in T4.

Step 4, on the other hand, was represented in a meeting composed of the two translators of T4 and the group of experts. This group of experts was composed of 16 professionals from the following areas: nursing, psychology, nutrition, pharmacy, medicine, social work, and gerontology. This step was essential to achieve language adequacy that aimed the improve the experience of the use with elderly patients who seek the emergency room.

Subsequently, the process of content validation was performed.^(20)^In the present study, the validation process was carried out by a panel of judges composed of two physicians, a physical therapist and a nurse (health professionals with hospital and academic experience in geriatrics and gerontology). The judges received a letter containing the questions of ISAR. They received the the necessary material for the content validation process. Thus, the judges received the research project of this study, the original manual of the ISAR tool, the adapted instrument, and the instructions for filling out the form for later calculation of the Content Validity Index (CVI). The CVI was considered the one corresponding to the accuracy rate of ≥80% for each item as a criterion for adequacy.^(18)^

It should also be noted that the process of cross-cultural adaptation should ensure equivalence between the original and the translated instrument in four areas: semantic, idiomatic, experimental, and conceptual.^(21)^

A pre-test, *i.e*., a pilot application of the Brazilian version of the ISAR was then carried out in order to verify the need for language adaptation of the instrument based on the understanding of elderly individuals, aged 65 years and older, and users of the emergency room of a teaching hospital. The sample age of 65 and older was chosen because the ISAR has been applied internationally within this age group.

The use of the ISAR was authorized by the authors of the tool and representatives of St. Mary’s Research Centre. This was approved by the Ethics Committees on Human Research of the School of Arts, Sciences and Humanities of *Universidade de São Paulo* (USP) #1,452,250, CAAE: 53923415.2.0000.5390, and of the *Hospital Universitário* of USP, # 1,457,299, CAAE: 53923415.2.3001.0076. All participants signed the Informed Consent Form, according to resolution 466/2012 of the National Health Council regarding Ethics in Research with Humans.

## RESULTS

In relation to question 1 of the ISAR, the word “emergency” was replaced by the word “emergency room”. In question 3 of the ISAR, the word “hospitalized” was changed to “inpatient”. Table 1 shows the original formulation of the tool, as well as the translations and consensus performed. Table 2 shows the last version of the ISAR questions sent for content validation.

**Table 1:** Presentation of the original formulation and translations of the tool Identification of Seniors at Risk

Original version - Question 1	Before the illness or injury that brought you to the emergency, did you need someone to help you on a regular basis?
Translation 1	*Antes da doença ou lesão que trouxe você à Emergência, você precisava de alguém para ajudá-lo regularmente?*
Translation 2	*Antes da doença ou lesão que trouxe você à Emergência, você precisava de alguém para ajudá-lo regularmente?*
Consensus	*Antes da doença ou lesão que trouxe você ao pronto socorro, você precisava de alguém para ajudá-lo regularmente?*
Original version - Question 2	In the last 24 hours, have you needed more help than usual?
Translation 1	*Nas últimas 24 horas, você precisou de mais ajuda do que usualmente (normalmente)?*
Translation 2	*Nas últimas 24 horas, você precisou de mais ajuda do que usualmente?*
Consensus	*Nas últimas 24 horas, você precisou de mais ajuda do que regularmente?*
Original version - Question 3	Have you been hospitalized for one or more nights during the past 6 months?
Translation 1	*Você foi hospitalizado por uma ou mais noites (mais de 24 horas) nos últimos 6 meses?*
Translation 2	*Você foi hospitalizado por uma ou mais noites nos últimos 6 meses?*
Consensus	*Você foi internado por uma ou mais noites nos últimos seis meses?*
Original version – Question 4	In general, do you have serious problems with your vision, that cannot be corrected by glasses?
Translation 1	*Em geral, você tem problemas sérios com sua visão, que não se corrigem com uso de óculos?*
Translation 2	*Em geral, você tem problemas sérios com sua visão, que não se corrigem com uso de óculos?*
Consensus	*Em geral, você tem problemas sérios com sua visão, que não se corrigem com uso de óculos?*
Original version – Question 5	In general, do you have serious problems with your memory?
Translation 1	*Em geral, você tem problemas sérios com sua (de) memória?*
Translation 2	*Em geral, você tem problemas sérios com sua memória?*
Consensus	*Em geral, você tem problemas sérios com sua memória?*
Original version – Question 6	Do you take six or more different medications every day?
Translation 1	*Você toma mais do que seis medicamentos diferentes todo dia (diariamente)?*
Translation 2	*Você toma seis ou mais medicamentos diferentes todos os dias?*
Consensus	*Você toma cinco ou mais medicamentos diferentes todos os dias?*

**Table 2:** Presentation of the latest version of the Identification of Seniors at Risk questions in Brazilian Portuguese

*1. Antes da doença ou lesão que trouxe você ao pronto-socorro, você precisava da ajuda de alguém regularmente?*
*2. Nas últimas 24 horas, você precisou de mais ajuda do que regularmente?*
*3. Você foi internado(a) por uma ou mais noites nos últimos 6 meses?*
*4. Em geral, você tem problemas sérios com sua visão, que não podem ser corrigidos com uso de óculos?*
*5. Em geral, você tem problemas sérios com sua memória?*
*6. Você toma cinco ou mais medicamentos diferentes todos os dias?*

Regarding the data from the content validation of the ISAR, the scores of the individual items were between three (agree) and four points (strongly agree).

For the instrument as a whole, an agreement rate of 0.90 was defined, using the criterion of “dividing the total number of items considered relevant by the judges by the total number of items”. In this sense, the agreement was 100% among the judges for the instrument, since all items were considered representative and relevant by the panel of judges that assessed the ISAR.

With these changes, the synthesis version of the ISAR was formalized and sent for back-translation, which showed no relevant difference when the version was compared to the original instrument. For this reason, it was considered that there were similarities of meaning and concept between the adapted and original versions, which resulted in the final version in Brazilian Portuguese (Table 3).

**Table 3:** Description of the final version of the Identification of Seniors at Risk tool in Brazilian Portuguese

*Questões*		*Uso exclusivo hospitalar*
*Antes da doença ou lesão que trouxe você ao hospital, você precisava de alguém para ajudá-lo regularmente?*	◻ *Sim*	1
	◻ *Não*	0
*Nas últimas 24 horas, você precisou de mais ajuda do que normalmente?*	◻ *Sim*	1
	◻ *Não*	0
*Você foi hospitalizado por uma ou mais noites nos últimos 6 meses?*	◻ *Sim*	1
	◻ *Não*	0
*Em geral, você tem problemas sérios com sua visão, que não se corrigem com uso de óculos?*	◻ *Sim*	1
	◻ *Não*	0
*Em geral, você tem problemas sérios com sua memória?*	◻ *Sim*	1
	◻ *Não*	0
*Você toma 6 ou mais medicamentos diferentes todos os dias?*	◻*Sim*	1
	◻ *Não*	0
	Total:	

*Por favor, responda sim ou não para cada uma dessas questões.*

To conclude the cross-cultural adaptation process, the ISAR was applied to 30 elderly participants, aged over 65 years. The language of the final version of the Brazilian ISAR tool was found to be adequate for the target population of the study.

## DISCUSSION

The results from the cross-cultural adaptation process of the ISAR tool showed the occurrence of some differences between the translations initially performed and the final consensus version. These differences are due to the need for language adaptation that goal was better cultural understanding.^(22)^

In question 1 of the ISAR, we perceived that the word “emergency room” is better understood by the elderly compared with the word “emergency”, and for this reason, we chose to change the word “emergency” to the compound word “emergency room”, preserving the meaning properties of both words.

In question 3 of the ISAR, it was found that the term “admitted” was better understood than the term “hospitalized”, because the first word is more used.

On the other hand, question 4 had changes during the content validation process, because it was discussed that the question had a double meaning. To solve this problem, the sentence was changed, aiming to make it impossible for the interviewees to understand the question twice, because of visual difficulties that cannot be corrected even with the use of glasses or corrective lenses.

There was also a change in question 6 of the ISAR. The original scale was designed considering the use of six or more medications. However, for the Brazilian reality, the expert group was based on the national standard established for polypharmacy and concluded that the number should be changed to five or more drugs.^(22)^

The etiology of polypharmacy is multifactorial. Chronic diseases typical of the aging process are one of the main elements for this manifestation.^(23)^Polypharmacy should be carefully approached and monitored by health professionals, since it is associated with increased risk and severity of adverse drug reactions (ADR), precipitated myocardial infarction, cumulative toxicity, medication errors, reduction of adherence to treatment, and increased morbidity and mortality. This practice is directly related to health care costs, such as visits to specialists, emergency care, and hospitalization.^(21)^

Still, research such as the Study on Health, Well-Being and Aging (SABE) evidenced the high prevalence of polypharmacy among the elderly population in the city of São Paulo (SP).^(24)^The SABE Study found that 31.5% of its sample used five or more prescribed or non-prescribed drugs. Also, another survey carried out by SABE, in the city of São Paulo, found that the prevalence of polypharmacy in 1,115 elderly individuals over 65 years of age was estimated in 36%.^(25)^

The vulnerability of the elderly to medication-related adverse events is quite high, which is due to the complexity of clinical problems, the need for multiple agents and the pharmacokinetic and pharmacodynamic changes inherent to aging. In this scenario, the great challenge for health professionals is to contribute to the promotion of the rational use of medicines. However, it should be emphasized the implementation of permanent education programs related to risk management, evidence-based error prevention barriers, and policies to encourage patient safety culture.^(24)^Therefore, the importance of actions that promote quality of life and health as a way to postpone or prevent situations like this from happening is once again evident.^(26)^

Thus, the suggestion of the group of specialists was accepted, since the quantity of drugs that defines polypharmacy in Brazil is lower than that stipulated in Canada.

Regarding the data from the content validation of the ISAR, the judges’ opinions considered its items representative and relevant for the purpose of the instrument. In this sense, there is 100% agreement between the judges for the instrument, because all items were considered representative and relevant by the panel of judges that assessed the ISAR.

The main limitation found in this stage of the study was related to the difficulty in achieving the validation of the ISAR, because the number of participants was lower than the sample size calculation. The reason for the insufficient sample size was caused by the lower demand of users in the emergency room during the data collection period of this study. The favorable result coming from the pilot application of the ISAR in the elderly in this study marked the possibility of further research to identify the psychometric properties of the ISAR in the Brazilian reality. Furthermore, studies that apply the ISAR in several populations of hospitalized elderly Brazilians are needed in order to identify the profile and demands of this population, according to the factors detected by the tool. It is also relevant to use as cut-off point ≥3 points to achieve better specificity.^(27)^

## CONCLUSION

The Identification of Seniors at Risk was translated and culturally adapted for use in Brazil. The semantic, idiomatic, cultural and conceptual equivalences obtained by a committee of judges in relation to the original Canadian version were attested. Our results showed that the Brazilian Identification of Seniors at Risk has verbal comprehension indexes and high content validity.

The adapted Brazilian version of the Identification of Seniors at Risk still needs to be analyzed for scale measurement properties with the purpose to provide better information reliability and, subsequently, to improve the use in clinical practice and better evaluation of adaptation aspects, to be used in elderly assisted in emergency rooms.

## References

[1] 1. Miranda GM, Mendes AC, Silva AL. Population aging in Brazil: current and future social challenges and consequences. Rev Bras Geriatr Gerontol. 2016;19(3):507-19.

[2] 2. Guerra IC, Ramos-Cerqueira AT. Risco de hospitalizações repetidas em idosos usuários de um centro de saúde escola. Cad Saude Publica. 2007;23(3):585-92. 10.1590/s0102-311x2007000300017 17334573

[3] 3. Oliveira RM, Leitão IM, Silva LM, Figueiredo SV, Sampaio RL, Gondim MM. Strategies for promoting patient safety: from the identification of the risks to the evidence-based practices. Esc Anna Nery. 2014;18(1):122-9.

[4] 4. Bertolucci PH, Brucki SM, Campacci SR, Juliano Y. O mini-exame do estado mental em uma população geral: impacto da escolaridade. Arq Neuro-Psiquiatr. 1994;52(1):1-7. 8002795

[5] 5. Boult C, Dowd B, McCaffrey D, Boult L, Hernandez R, Krulewitch H. Screening elders for risk of hospital admission. J Am Geriatr Soc. 1993;41(8):811-7. 10.1111/j.1532-5415.1993.tb06175.x 8340558

[6] 6. Boult C, Wieland GD. Comprehensive primary care for older patients with multiple chronic conditions: “Nobody rushes you through”. JAMA. 2010;304(17):1936-43. 10.1001/jama.2010.1623 21045100

[7] 7. Nunes BP, Soares MU, Wachs LS, Volz PM, Saes MO, Duro MS, et al. Hospitalização em idosos: associação com multimorbidade, atenção básica e plano de saúde. Rev Saude Publica. 2017;51:43. 10.1590/S1518-8787.2017051006646 PMC5433790 28492761

[8] 8. Yao JL, Fang J, Lou QQ, Anderson RM. A systematic review of the identification of seniors at risk (ISAR) tool for the prediction of adverse outcome in elderly patients seen in the emergency department. Int J Clin Exp Med. 2015; 8(4):4778-86. Review. PMC4483958 26131052

[9] 9. Asomaning N, Loftus C. Identification of seniors at risk (ISAR) screening tool in the emergency department: implementation using the plan-do-study-act model and validation results. J Emerg Nurs. 2014;40(4):357-64.e1. 10.1016/j.jen.2013.08.014 24144796

[10] 10. Galvin R, Gilleit Y, Wallace E, Cousins G, Bolmer M, Rainer T, et al. Adverse outcomes in older adults attending emergency departments: a systematic review and meta-analysis of the Identification of Seniors At Risk (ISAR) screening tool. Age Ageing. 2017;46(2):179-86. Review. 10.1093/ageing/afw233 27989992

[11] 11. Singler K, Heppner HJ, Skutetzky A, Sieber C, Christ M, Thiem U. Predictive validity of the identification of seniors at risk screening tool in a German emergency department setting. Gerontology. 2014;60(5):413-9. 10.1159/000358825 24969966

[12] 12. Rosted E, Schultz M, Dynesen H, Dahl M, Sørensen M, Sanders S. The identification of seniors at risk screening tool is useful for predicting acute readmissions. Dan Med J. 2014;61(5):A4828. 24814738

[13] 13. Salvi F, Belluigi A, Cherubini. Predictive validity of different modified versions of the identification of seniors at risk. J Am Geriatr Soc. 2013;61(3):462-4. 10.1111/jgs.12130 23496185

[14] 14. Salvi F, Morichi V, Grilli A, Lancioni L, Spazzafumo L, Polonara S, et al. Screening for frailty in elderly emergency department patients by using the Identification of Seniors At Risk (ISAR). J Nutr Health Aging. 2012; 16(4):313-8.10.1007/s12603-011-0155-922499448

[15] 15. Aldeen AZ, M, LA, , Geriatric emergency department innovations: preliminary data for the geriatric nurse liaison model. J Am Geriatr Soc. 2014;62(9):1781-5. 10.1111/jgs.12979 25112656

[16] 16. Beaton DE, Bombardier C, Guillermin F, Ferraz MB. Guidelines for the process of cross-cultural adaptation of self-report measures. Spine (Phila Pa 1976). 2000;25(24):3186-91. Review. 10.1097/00007632-200012150-00014 11124735

[17] 17. Guillemin F. Cross-cultural adaptation and validation of health status measures. Scand J Rheumatol.1995;24(2):61-3. 10.3109/03009749509099285 7747144

[18] 18. Ferreira L, Neves AN, Campana MB, Tavares MC. Guia da AAOS/IWH: sugestões para adaptação transcultural de escalas. Aval Psicol. 2014;13(3):457-61.

[19] 19. Raymundo VP. Construção e validação de instrumentos: um desafio para a psicolinguística. Letra Hoje. 2009;44(3):86-93.

[20] 20. Alexandre NM, Coluci MZ. Validade de conteúdo nos processos de construção e adaptação de instrumentos de medidas. Cien Saude Colet. 2011;16(7):3061-8. 10.1590/s1413-81232011000800006 21808894

[21] 21. Pedroso RS, Oliveira MS, Araujo RB, Moraes JF. Tradução, equivalência semântica e adaptação cultural do Marijuana Expectancy Questionnaire (MEQ). Psico USF. 2004;9(2):129-36.

[22] 22. Secoli SR. Polifarmácia: interações e reações adversas no uso de medicamentos por idosos. Rev Bras Enferm. 2010;63(1):136-40. 10.1590/s0034-71672010000100023 20339769

[23] 23. Lebrão ML, Laurenti R. Saúde, bem-estar e envelhecimento: o estudo sabe no Município de São Paulo. Rev Bras Epidemiol. 2005;8(2):127-41.

[24] 24. Carvalho MF, Romano-Lieber NS, Bergsten-Mendes G, Secoli SR, Ribeiro E, Lebrão ML, et al. Polypharmacy among the elderly in the city of São Paulo, Brazil – SABE Study. Rev Bras Epidemiol. 2012;15(4):817-27. 10.1590/s1415-790x2012000400013 23515777

[25] 25. Reis MA, Gabriel CS, Zanetti AC, Bernardes A, Laus AM, Pereira LR. Medicamentos potencialmente perigosos: identificação de riscos de prevenção de erros em terapia intensiva. Texto Contexto Enferm. 2018;27(2):e5710016.

[26] 26. Yao J, Fang J, Lou QQ, Anderson RM. A systematic review of the identification of seniors at risk (ISAR) tool for the prediction of adverse outcome in elderly patients seen in the emergency department. Int J Clin Exp Med. 2015;8(4):4778-86. Review. PMC4483958 26131052

[27] 27. Warburton RN, Parke B, Church W, McCusker J. Identification of seniors at risk: process evaluation of a screening and referral program for patients aged > or =75 in a community hospital emergency department. Int J Health Care Qual Assur Inc Leadersh Health Serv. 2004;17(6):339-48. 10.1108/09526860410557598 15552389

